# Measuring plasma levels of three microRNAs can improve the accuracy for identification of malignant breast lesions in women with BI-RADS 4 mammography

**DOI:** 10.18632/oncotarget.20806

**Published:** 2017-09-11

**Authors:** Julia Alejandra Pezuk, Thiago Luiz Araujo Miller, José Luiz Barbosa Bevilacqua, Alfredo Carlos Simões Dornellas de Barros, Felipe Eduardo Martins de Andrade, Luiza Freire de Andrade e Macedo, Vera Aguilar, Amanda Natasha Menardo Claro, Anamaria Aranha Camargo, Pedro Alexandre Favoretto Galante, Luiz F.L. Reis

**Affiliations:** ^1^ Hospital Sírio-Libanês, São Paulo, Brazil; ^2^ Departamento de Bioquímica, Instituto de Química, Universidade de São Paulo, São Paulo, Brazil; ^3^ Current address: SulAmerica, São Paulo, Brazil

**Keywords:** micro RNAs, breast cancer, BI-RADS, blood plasma, machine learning

## Abstract

A BI-RADS category of 4 from a mammogram indicates suspicious breast lesions, which require core biopsies for diagnosis and have an approximately one third chance of being malignant. Human plasma contains many circulating microRNAs, and variations in their circulating levels have been associated with pathologies, including cancer. Here, we present a novel methodology to identify malignant breast lesions in women with BI-RADS 4 mammography. First, we used the miRNome array and qRT-PCR to define circulating microRNAs that were differentially represented in blood samples from women with breast tumor (BI-RADS 5 or 6) in comparison to controls (BI-RADS 1 or 2). Next, we used qRT-PCR to quantify the level of this circulating microRNAs in patients with mammograms presenting with BI-RADS category 4. Finally, we developed a machine learning method (Artificial Neural Network - ANN) that receives circulating microRNA levels and automatically classifies BI-RADS 4 breast lesions as malignant or benign. We identified a minimum set of three circulating miRNAs (miR-15a, miR-101 and miR-144) with altered levels in patients with breast cancer. These three miRNAs were quantified in plasma from 60 patients presenting biopsy-proven BI-RADS 4 lesions. Finally, we constructed a very efficient ANN that could correctly classify BI-RADS 4 lesions as malignant or benign with approximately 92.5% accuracy, 95% specificity and 88% sensibility. We believe that our strategy of using circulating microRNA and a machine learning method to classify BI-RADS 4 breast lesions is a non-invasive, non-stressful and valuable complementary approach to core biopsy in women with BI-RADS 4 lesions.

## INTRODUCTION

Breast cancer (BC) is the second most common malignancy in women [1]. As BC patients’ life quality and survival decrease significantly with late BC diagnosis, early tumor detection is critical to improve the disease outcome [1]. Currently, mammography is the preferred and most used method for early BC detection. Despite its modest sensitivity (approximately 80%) and specificity (approximately 85%), it is still a valuable method because its assessment has been associated with a reduction in BC morbidity and mortality worldwide.

The *Breast Imaging and Reporting Data System* (BI-RADS^®^) is a classification system used by radiologists to sort mammography results into categories numbered from 0 through 6 [2]. The classification depends on radiological features such as breast mass, calcification, architectural distortion, asymmetries and intramammary lymph nodes, which demonstrate good correlation with the likelihood of the absence or presence of BC. Category 0 is for incomplete results, and further radiological evaluation is needed. Categories 1 and 2 correspond to non-malignant breast lesions. Category 3 is probably benign lesions. BI-RADS category 5 corresponds to highly suggestive malignancy, with an over 95% of likelihood, and category 6 is for proven malignancy [2]. However, the most uncertain lesions are those of BI-RADS category 4, which are divided into three sub categories according to the probability of malignancy: 4a, probability of malignancy 2–10%; 4b, probability of malignancy 10–50%; and 4c, probability of malignancy 50–95%. Currently, all BI-RADS category 4 warrant further evaluation [3].

Although BI-RADS category 4 can be subdivided according to its likelihood of cancer, 90% of women with BI-RADS 4 lesions are submitted to core biopsies to confirm their breast lesion classification [3]. Based on our experience and on the data from the literature, only 30% of BI-RADS 4 lesions are confirmed as malignant. Therefore, most women with BI-RADS category 4 lesions are submitted to an unnecessary invasive and stressful approach. In this scenario, the identification of non-invasive biomarkers capable of discriminating malignant from benign BI-RADS category 4 lesions would have a positive impact on patient life quality and BC diagnosis.

MicroRNAs (miRNAs) are small non-coding RNAs with heavy involvement in gene expression regulation in plant and animals [4]. Alterations in miRNA levels have been associated with different pathologies, including cancer [5, 6]. It is known that miRNAs can be released and detected in the blood and that variations in their circulating levels have been associated with different pathological and physiological conditions over the past decade [7].

Circulating biomarkers present great promise as non-invasive markers to improve cancer diagnosis and to monitor therapy response and disease progression [8]. Among all circulating molecules, mature miRNAs are ‘gold standard’ biomarkers because they are highly stable, without major post transcriptional variations, short in length and can be measured using relatively simple and cost-effective methodologies. The potential use of circulating miRNAs as biomarkers has been described for many cancer types, including BC [9]. For example, alterations in miRNA circulating levels have been used as a screening tool for BC diagnosis [10, 11], for molecular subtype classification [12] and for metastasis detection [13].

Here, we have made a hypothesis-generating study, in which we developed a non-invasive method to accurately classify BI-RADS 4 breast lesions as malignant or benign. Our method combines two cutting edge strategies, the quantification of circulating miRNA levels in the patient blood and a machine learning method (Artificial Neural Network - ANN) to classify BI-RADS category 4 breast lesions as malignant or benign. For our set of approximately 60 BI-RADS category 4 lesions, our method presented an accuracy, sensitivity and specificity of approximately 90% to classify BI-RADS category 4 lesions as malignant or benign. Thus, we here present a novel noninvasive, low stress and potentially cost effective strategy to classify BI-RADS category 4 lesions.

## RESULTS

### Identifying circulating miRNAs differentially represented in breast cancer

**Figure 1:**
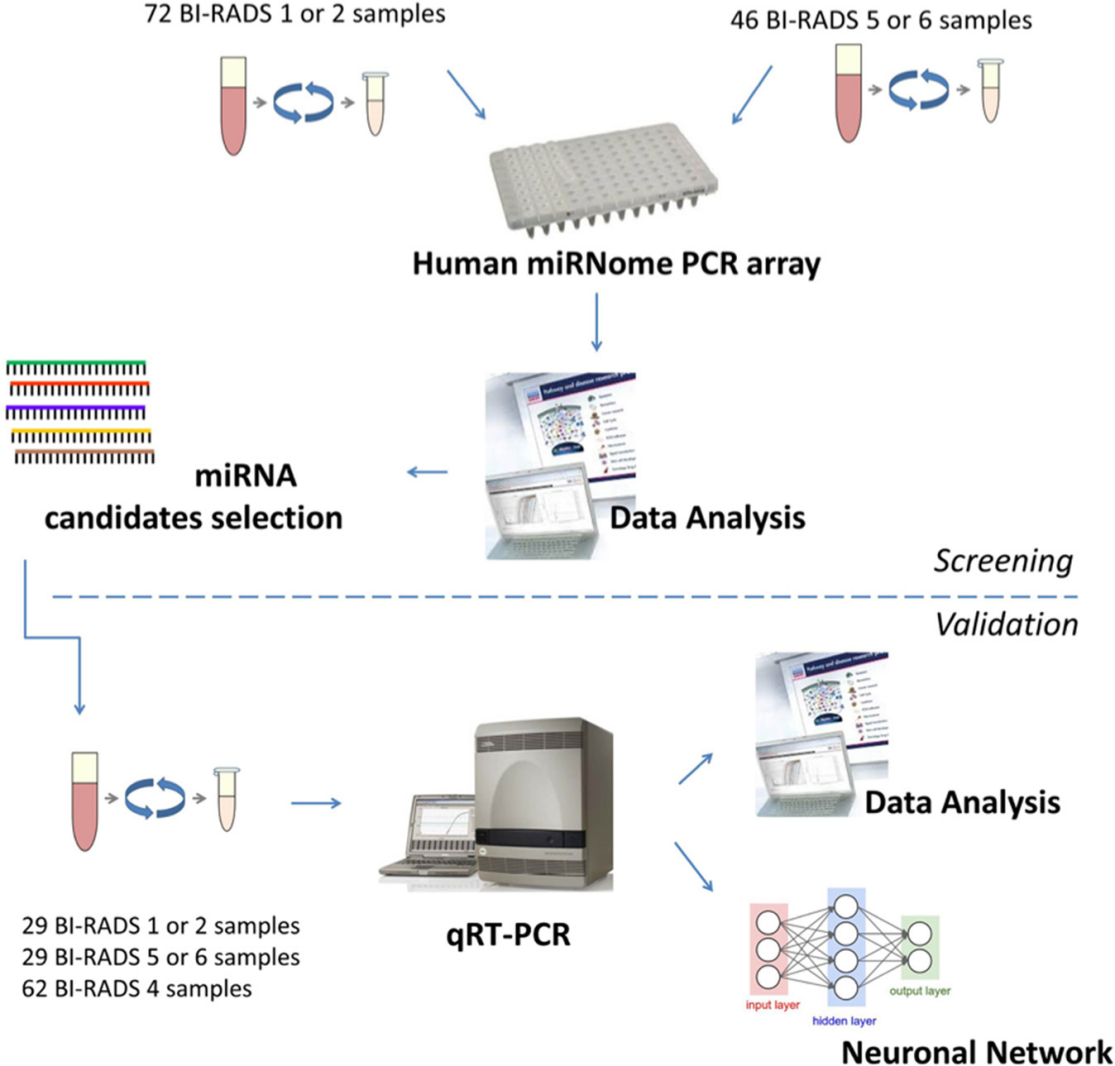
Experimental strategy The screening cohort (*n* = 118) contains 72 controls (BI-RADS 1 or 2) and 46 cancer samples (BI-RADS 5 or 6). The validation cohort (*n* = 120) contain 29 cancer samples, 29 control samples and 62 test samples (BI-RADS 4).

Using a large-scale platform (miRNome PCR array), we compared the levels of 1,805 miRNAs in the plasma of 46 breast cancer patients (breast lesion with BI-RADS categories 5 or 6 from mammography) versus 72 control patients (breast lesions with BI-RADS categories 1 or 2 from mammography). The global Ct mean of all circulating miRNAs in all samples was used as the circulating level calibrator, Figure 1.

**Figure 2:**
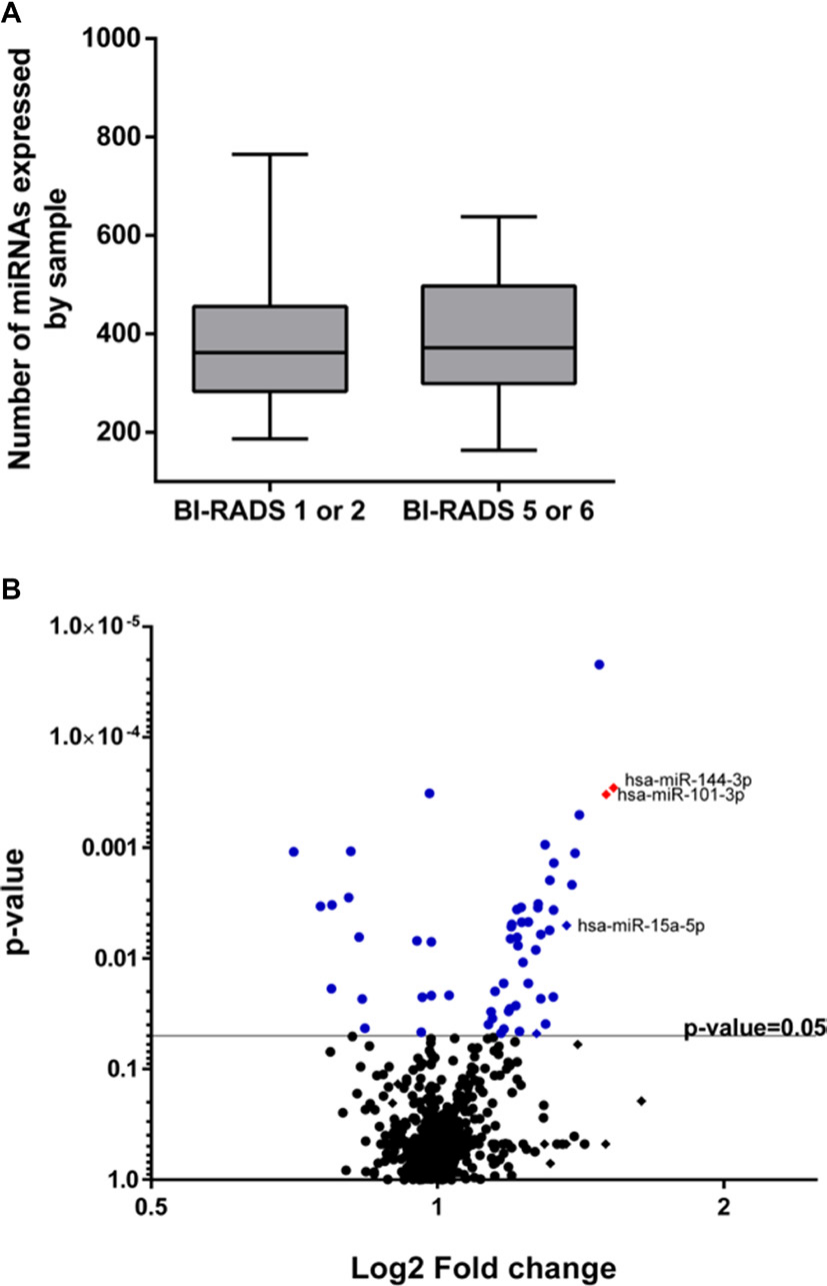
miRNAs detected and differentially represented (**A**) Box plot showing the number of circulating miRNAs detected in controls (BI-RADS category 1 and 2) and breast cancer samples (BI-RADS category 5 and 6). (**B**) Volcano plot comparing circulating miRNA levels between control and BC. In blue and red are all 57 miRNAs differentially represented (*p*-value < 0.05) in BC samples. Red miRNAs also have a log2 fold change > 1.5.

First, we detected a mean of ~20% of all known miRNAs per sample, and no difference was found in terms of the number of circulating miRNAs in controls and BCs (Figure 2A). Next, we searched for differentially represented circulating miRNAs between BI-RADS category 1 and 2 (controls) versus BI-RADS category 5 and 6 (breast cancer) lesions. We found a total of 57 circulating miRNAs differentially represented (adjusted *p*-value < 0.05; Figure 2B), 46 of which were over and 9 of which were under represented in the plasma of patients with breast cancer (Figure 2B). A full list of those differentially represented miRNAs are in Supplementary Table 1.

### Identifying a minimum set of circulating miRNAs over represented in breast cancer

To be clinically feasible, a short list of biomarkers is the aim to reduce the handling time, amount of material and costs. Therefore, we aimed to reduce our list of 57 candidates to a minimum set of circulating miRNAs differentially represented in BC versus control. Then, based on the expression ratio (high), *p*-value (low) and literature support (related to BC), we selected 12 miRNAs to be further tested in an independent set of 58 plasma samples, 29 controls (breast lesion classified as BI-RADS 1–2) and 29 BC (BI-RADS 5–6) (Supplementary Table 2).

**Figure 3:**
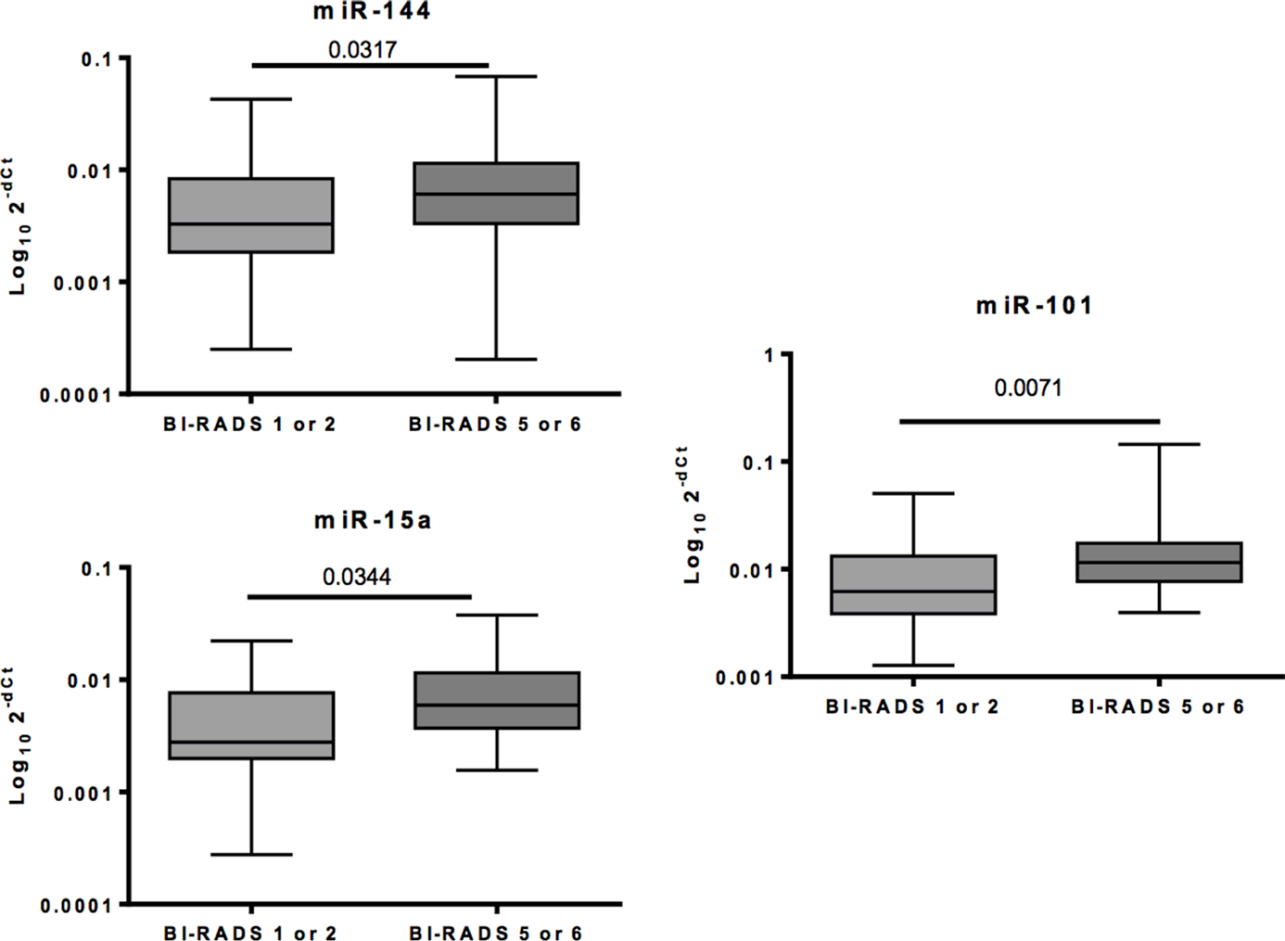
MiRNAs differentially represented in BC versus controls

Among all 12 circulating miRNAs tested in the second cohort, three miRNAs (miR-15a, miR-144 and miR-101) emerged as differentially represented in BC, with a fold change over 1.5x and a *p*-value < 0.05 (Mann-Whitney test) (Figure 3). All other circulating miRNAs were not detected or did not present differential levels in this independent set of BCs versus controls lesions (Supplementary Figure 1 and Supplementary Table 2). For normalization purposes, we included miR-16, RNU6-b, cel-miR-39 and the two most stable miRNAs (miR-3173 and miR-1280) as calibrator miRNAs (Supplementary Table 3). Using this additional step, we were therefore able to reduce the list of circulating miRNAs differentially represented in the plasma of controls (women with BI-RADS category 1–2 lesions) versus BC (women with BI-RADS category 5–6 lesions) to a minimum set of three miRNAs: miR-15a, miR-144 and miR-101.

### miRNA levels in BI-RADS category 4 breast lesions and ANN lesion classification

As BI-RADS category 4 requires core biopsies for diagnosis confirmation, we obtained the plasma of 62 women with biopsy-proven breast lesions already classified as malignant (67% of our samples) or benign (33% of our samples). Then, we quantified the level of the three circulating miRNAs (miR-15a, miR-144 and miR-101) in the plasma of these women. Fortunately, the three tested miRNAs presented a usable 2^-ΔCT^ level (see Methods) for 97% of samples (Supplementary Figure 1).

**Figure 4:**
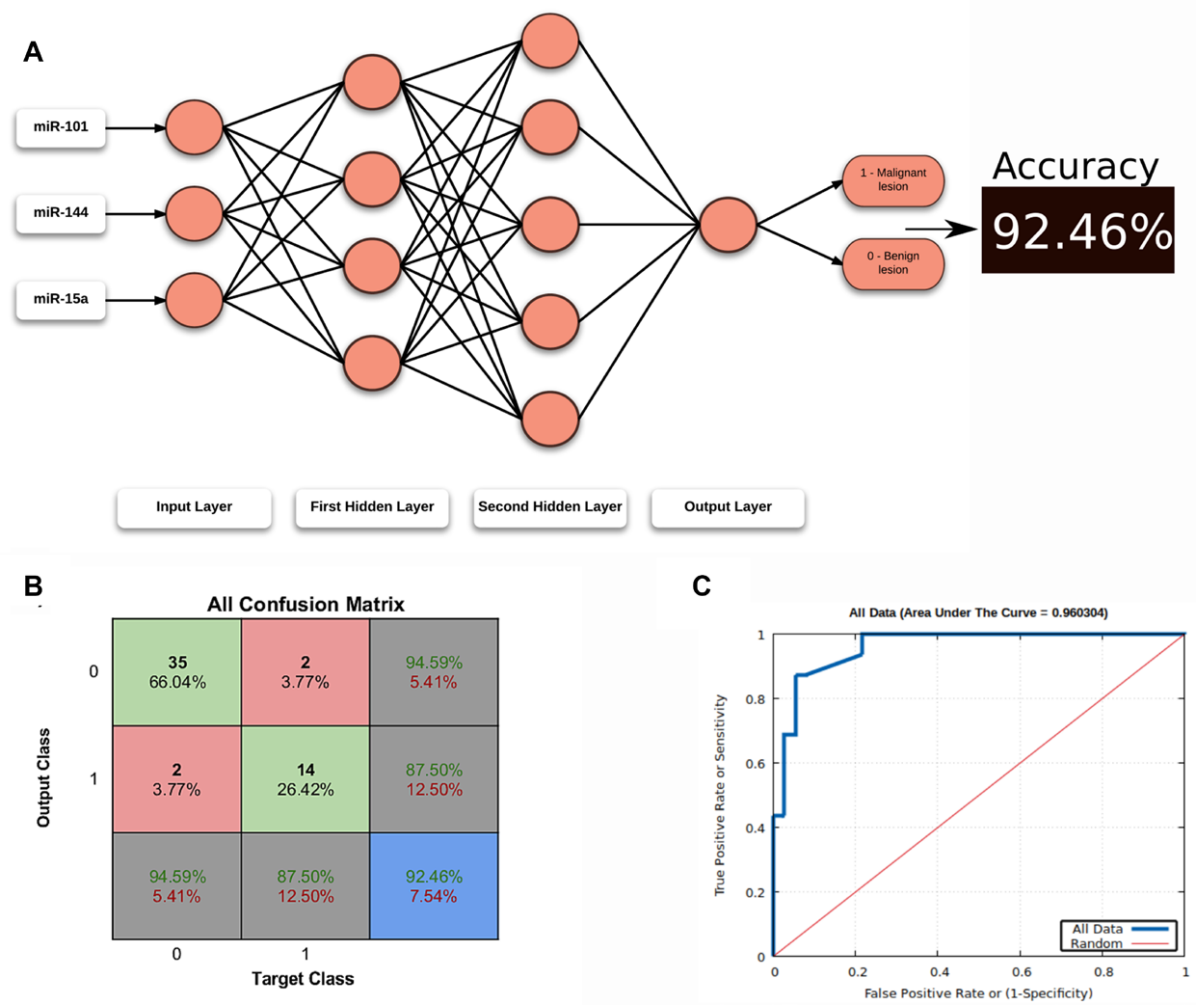
ANN topology and classification (**A**) Structure of the best ANN topology. (**B**) Confusion matrix summarizing all data used in ANN. In the blue cell: red font, percentage of misclassification, green font, percentage of correct classifications. (**C**) ROC Curve for all data with the respective Area Under the Curve (AUC).

We next developed an ANN, a machine learning method, to receive the miRNA levels, process them and classify each breast lesion as benign or malignant. Defining the network topology is an important step to find the best ANN for each approach. In our case, we tested several topologies, but a network with 4 neurons in the first hidden layer, 5 neurons in the second hidden layer and 1 neuron in the output layer, with all layers having Sigmoid Symmetric transfer functions, was our best ANN topology to classify our set of BI-RADS category 4 lesions. The training function selected was RPROP (resilient backpropagation algorithm; check Methods, Supplementary Figure 2 and Supplementary Tables 5–6 for details) (Figure 4A).

To find the best ANN for our sample set classification, we randomly split (10,000×) the circulating miRNA levels into training (60%), validation (20%) and test (20%) groups. To avoid any model overfitting, we implemented an early stopping regularization during the training step, and we also used a multilayer feedforward ANN training with the backpropagation algorithm (Supplementary Figure 3).

In the end, we obtained a very efficient ANN for classifying our BI-RADS category 4 lesions. Using the biopsy proven lesions as positive controls, our ANN correctly classified 92.46% (accuracy) of all lesions (Figure 4B), with a sensitivity of 100% and specificity of 90.00% for the test set and sensitivity of 87.50% and specificity of 94.59% for all breast lesions (Figure 4C). Therefore, the positive and negative likelihood ratios were 16.2 and 0.13, respectively.

## DISCUSSION

Mammography screening has drastically improved early BC diagnosis and the BC survival rate. However, despite this accessibility, it is an unpleasant procedure and has limitations due to ionizing radiation, breast density, technician inexperience and inaccuracy that lead to false results in over 10% of cases. Moreover, patients with suspicious lesions (BI-RADS category 4) usually need an additional and invasive procedure (a core biopsy) to confirm the diagnosis of BC. Here, we present a novel non-invasive methodology to identify malignant breast lesions in women with BI-RADS 4 mammography.

Circulating miRNAs are stable and are free or associate with proteins [14], and therefore the possibility of using circulating miRNAs as biomarkers has already been proposed for several pathologies [10, 11, 15]. It is well known that each cell type liberates miRNAs to its surroundings and that those cell-free molecules can be detected in several biological fluids, including serum and plasma. Circulating miRNAs can be more effective than other biomarkers once their expression has not shown correlation with clinic-pathological features such as hormone receptors, HER expression, Ki67, lymph node status, tumor size or age [10] and they are stable molecules with low post transcriptional variations that are potentially individually related. Circulating miRNAs have been largely explore in breast cancer, and a role on prognosis, diagnosis and as therapy response monitors have been proposed [16], however the relation with BI-RADS has not been explored yet.

An important limitation to studying circulating miRNAs is to choose the right normalization method [17, 18]. We systematically tested for combinations of two circulating miRNAs, the most stable miRNAs during the screening phase (miR-3173) and the spike in cel-miR-39. Therefore, we believed that our strategy of quantifying the level of circulating miRNAs in the plasma of patients presenting benign or malignant breast lesions are reduced in terms of the side and noise effects caused by experimental handling and/or individual patient backgrounds.

Machine learning algorithms, powered by advances in computation, data quality and large-scale data generation, have recently been shown to exceed human performance in several areas, including pattern recognition and visual tasks. A fruitful area for the application of machine learning methods is medicine. The analyses of several aspects of patient information and the accurate identification of a disease is one of the most challenging tasks for physicians. On the other hand, machine learning methods, whether well-constructed and trained, are powerful strategies for the analyses of complex datasets and effectively identify a pattern, such as a suspicion of cancer. The ANN, a Machine Learning Method developed here, accurately classifies BI-RADS category 4 breast lesions. Moreover, once created, ANN are not computationally intense and could be executed on a standard PC or even in a web tool, where the user could input the level of the circulating miRNAs and easily obtain the breast lesion classification between malignant and benign. However, further prospective analyses are necessary to evaluate the performance of our ANN in a clinic routine to confirm its very high accuracy, sensitivity and specificity. We are expanding its application to some of our patients presenting BI-RADS category 4.

Despite facilities performing percutaneous imaging-guided biopsies in patients with breast lesions classified as BI-RADS category 4, it is still an invasive procedure that requires a medical doctor and appropriate facilities, with some potential side effects and a relatively high cost. On the other hand, measuring alterations in circulating miRNAs levels is a non-invasive method that requires only blood sampling, a technician to perform qRT-PCR–similarly to other blood tests routinely applied today–and a professional to fill a table with three values of the circulating miRNAs level for each sample. The ANN will automatically classify the breast lesion in malignant or benign and return the result.

The three miRNAs (miR-15a, miR-144 and miR-101) emerged here have already been described in BC, but neither of them have been related to BI-RADS nor their circulating level used together to distinguish benign from malignant breast lesions. For example, differential expression of miR-15a was observed in BC and it was potentially correlated to a bad prognosis factor [19]. MiR-144 has been proposed as a BC marker, once that it is, in potential, an important regulator of tumorigenesis and tumor progression with a putative role on cell migration and invasion [20]. MiR-101 was also described as a good therapeutic target for BC and changing on its expression level seems to be related to proliferation, invasion and autophagy, and apoptosis in BC cells [21].

In summary, here we propose a non-invasive methodology to distinguish benign from malignant breast lesions previously classified as BI-RADS category 4 in mammography. Our strategy combines two cutting edge methodologies, the quantification of a minimum set of circulating miRNAs and a learning machine technique (ANN) to accurately classify BI-RADS category 4 breast lesions between benign and malignant. After prospective validation, our methodology should be a valuable approach to support or even replace (in the future) biopsy in women with BI-RADS category 4 lesions, as well as be used (in potential) as an additional approach to reduce false-negative biopsy results in BC.

## MATERIALS AND METHODS

### Patient cohort

A total of 238 blood samples were collected from women undergoing mammography at Hospital Sírio Libanês in the period between 2013 and 2015. Blood samples were collected (patient blood withdrawn) before mammography and core biopsy. All women provided written informed consent for collection and molecular analysis of blood specimens. This study was approved by the Hospital Sírio Libanês Ethics Committee (Study #2013–03). The sample characteristics are summarized in Table 1 (for more details see Supplementary Table 4). Control patients have at least one previous mammogram with normal results and no history of cancer, while cancer patients did not have previous cancer nor received treatment before sample collection.

**Table 1:** Patient characteristics for the screening (*n* = 118) and validation and testing (*n* = 120) phases of the study

Characteristics	Control patients (BI-RADS 1 or 2) - screening (*n* = 72)	Breast cancer patients (BI-RADS 5 or 6) - screening (*n* = 46)	Control patients (BI-RADS 1 or 2) - validation (*n* = 29)	Breast cancer patients (BI-RADS 5 or 6) - validation (*n* = 29)	Test patients (BI-RADS 4) - testing (*n* = 62)
**Median age**	51.6 ± 11.3	58.1 ± 11.5	54.28 ± 9.99	56.73 ± 13.45	51.29 ± 11.96
**IMC**	26.25 ± 4.5	27.73 ± 5.86	26.81 ± 4.2	25.67 ± 4.54	27.05 ± 4.72
**Immunohistochemistry (%)**					
Without hormonal receptors	-	6.52	-	6.9	-
With hormone receptors	-	8.7	-	3.45	-
Luminal A	-	17.39	-	13.79	-
Luminal B	-	50	-	68.97	-
Triple negative	-	4.35	-	-	-
Not specify	-	13.04	-	3.45	-
HER-2	-	-	-	3.45	-
**Metastasis (%)**					
Negative Lymph node	-	67.39	-	44.83	-
Positive lymph node	-	21.74	-	37.93	-
Not specified	-	10.87	-	17.24	-

### Sample processing

We used plasma to avoid coagulation, a process that can trigger an activation cascade that will release cell molecules causing variation in circulating miRNAs levels [22]. Blood was collected in EDTA tubes and processed within the first 2 hours after collection to guarantee plasma quality. Briefly, to separate plasma, blood samples were centrifuged at 1,900 g for 10 minutes at 4°C. Next, to remove debris, plasma was centrifuged at 16,000 g for 10 minutes at 4°C. The pellet was discarded, and plasma was aliquoted and stored at −80°C (protocol modified from [11]). To evaluate blood cell hemolysis, we measured the plasma absorbance at 414 nm (ABS_414_). ABS_414_ was quantified using the NanoVue (GE Healthcare Life Sciences^®^). Samples with absorbance values lower than 0.2 were considered non-hemolytic as described by Kirschner et al. [23]. All samples analyzed in this study were below the cutoff of 0.2.

### Isolation of miRNA from plasma

RNA was isolated following a phenol-free protocol. Briefly, proteins were first precipitated using a metal cation, and then, RNA present in the supernatant was bound to an RNeasy column and washed with diluted buffers RWT and RPE and subsequently with ethanol prior to drying and elution with RNAse-free water. cDNA was pre-amplified for 12 cycles to guaranty the detection of circulating miRNAs by using the miScript PreAMP PCR Kit (Qiagen^®^).

### Sample quality

Several controls were used to determine the RNA sample quality. To monitor variation during sample preparation, *C. elegans* miR-39 mimic was spiked into each sample. MiScript PCR Controls were included to quantify a panel of 5 snoRNAs (SNORD61, SNORD68, SNORD72, SNORD95 and SNORD96A) and the snRNA RNU6B (RNU6–2), all of which have stable levels in plasma. Additionally, miRNA reverse transcription control RNA (miRTC) and positive PCR controls (PPC) were used to monitor variables that may inhibit reverse transcription or amplification. To evaluate sample quality, we calculated the difference as (Average Ct value of RTC) – (Average Ct value of PPC), with values lower than 7 indicating no inhibition. Additionally, PPC Ct values of 19 ± 2 indicate no contaminants or inhibition.

### MiRNome array

Circulating miRNA profiling was carried out using the miRNome array v18.0 from Qiagen^®^ in the 384-well format (MIHS-3218Z v18.0). These arrays measure the expression of 1,805 miRNAs plus controls. This array contains a panel of 384 primer sets: 370 relevant miRNA genes plus five housekeeping genes and three RNA and PCR quality controls. The detection was performed using SYBR^®^ Green real-time RT-PCR.

### Validation of selected marker candidates

To validate the differences in miRNA circulating levels, total RNA was extracted from 200 μl of plasma using the miRNeasy serum/plasma kit (Qiagen^®^). Denaturation and phase separation was carried out using Qiazol Lysis Reagent^®^ according to the fabricant's protocol. Before phase separation, 3.5 μl of *C. elegans* miR-39 was added at a concentration of 1.6 × 10^8^ copies/μl. Next, the aqueous phase was transferred into another tube, ethanol was added and processing was performed using the QIAcube from Qiagen^®^. MiRNAs were eluted in 16 μl of RNAse-free water and quantified using the NanoDrop 2000 (Thermo Scientific^®^). Then, miRNAs were retro-transcribed into cDNA using the miScript^®^ II RT kit from Qiagen^®^ according to the manufacturer's instruction. The 5x miScript HiSpect buffer was used to obtain mature miRNAs. cDNA was pre-amplified for 12 cycles using the miScript^®^ PreAMP PCR kit, and real time PCR was carried out using miScript SYBR^®^ Green PCR kit with the miScript HiSpec buffer using Qiagen^®^ kits. MiRNA-specific primers for all candidates were purchased from Qiagen^®^, and qRT-PCR was performed using the Applied Biosystems 7900HT thermocycler. Analyses were carried out using the 2^-ΔCT^ formula for each sample using the mean of cel-miR-39 and miR-3173 for normalization.

### Artificial neural network

ANN is a machine learning method that is widely used for pattern recognition and data classification. This method receives an input, splits it into three sets (training, validation and test), self learns from the data and releases an ANN that is able, for example, to classify an independent set of elements. Working with ANN requires properly pre-processed data to be fed into the algorithm, which consists of, for example, standardization and removing outlier values. This step should be performed to avoid range scaling problems, thereby improving the probability of a good ANN performance. Here, the circulating microRNA level (2^-ΔCT^) obtained by qRT-PCR underwent an outlier filter, in which some measurements were removed following the Tukey rules on interquartile range (IQR). All circulating miRNA levels between Q1 - 1.5 * IQR and Q3 + 1.5 * IQR were kept to carry out our ANN modeling. The selected data were standardized for each microRNA to have a centralized set, with zero (central point), +1 (maximum possible value) and -1 (minimum possible value). Next, we applied in-house software written in the ANSI-C programming language and using the Fast Artificial Neural Network library (FANN [24], version 2.2.0) to find the ANN optimal topology capable to classify BI-RADS category 4 breast lesions as malignant or benign. Briefly, the algorithm consists of testing various ANN combinations to find the best network topology. Each ANN combination was thus performed in a stochastic way, with random selection of parameters (such as the number of neurons per layer) and random splits of the whole data in three sets: one for training, one for validation and one for testing. When the combination was defined, the parameters were used to create a multilayer feedforward ANN, training it with the backpropagation algorithm; to avoid model overfitting, we implemented early stopping regularization during training. The stopping step was defined by comparing the errors, where if the obtained error is lower than the lowest error so far, then the previous best ANN is replaced by the current one (Supplementary Figure 3). For quantify the ANN classification efficiency, we defined their accuracy (true results / total number of cases), sensitivity (true positive / positive) and specificity (true negative / negative).

### Statistical analysis

Statistical analyses for selecting the set of miRNAs with significant differences in their circulating levels between controls and breast cancer samples were performed by the Qiagen Data Analysis center. The data were normalized to the global mean of the circulating miRNA levels. The Normfinder^®^ software was used to determine calibrators’ genes on the validation phase to choose the best combination from candidate housekeeping genes for the validation phase. The Mann-Whitney statistic was calculated using GraphPad software version 6.0 to analyze circulating levels. The correlation between miRNA circulating levels and age, IMC and breast feeding was analyzed using the Pearson formula in GraphPad. All tests were carried out for an α value of 0.05.

## SUPPLEMENTARY MATERIALS FIGURES AND TABLES


