# Supplementary material for: Measuring plasma levels of three microRNAs can improve the accuracy for identification of malignant breast lesions in women with BI-RADS 4 mammography
Source: Oncotarget. 2017 Sep 11;8(48):83940–8. doi: 10.18632/oncotarget.20806 (PMC5663566; doi:10.18632/oncotarget.20806)
Supplement: Supplementary file 1 [file oncotarget-08-83940-s001.pdf]

Measuring plasma levels of three microRNAs can improve the accuracy for identification of malignant breast lesions in women with BI-RADS 4 mammography

SUPPLEMENTARY MATERIALS

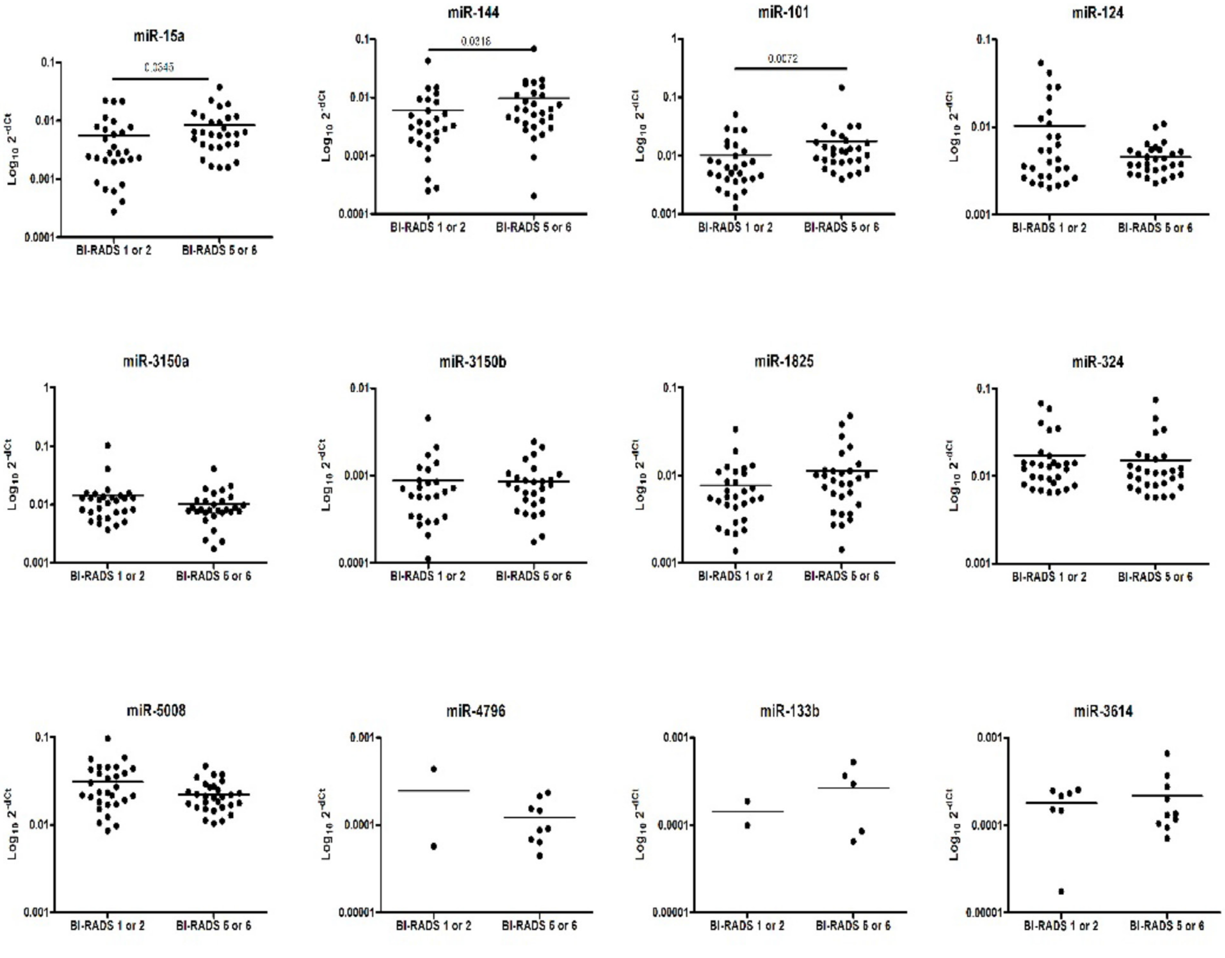

Supplementary Figure 1: MiRNAs Differential expressed in plasma between BI-RAS 1 or 2 and BI-RADS 5 or 6 patient samples. Relative expression of select miRNAs in 29 control plasma samples (BIRADS 1 or 2) and 29 cancer plasma samples (BI-RDAS 5 or 6). $P < 0.05$ using Mann Whitney was considered significant.

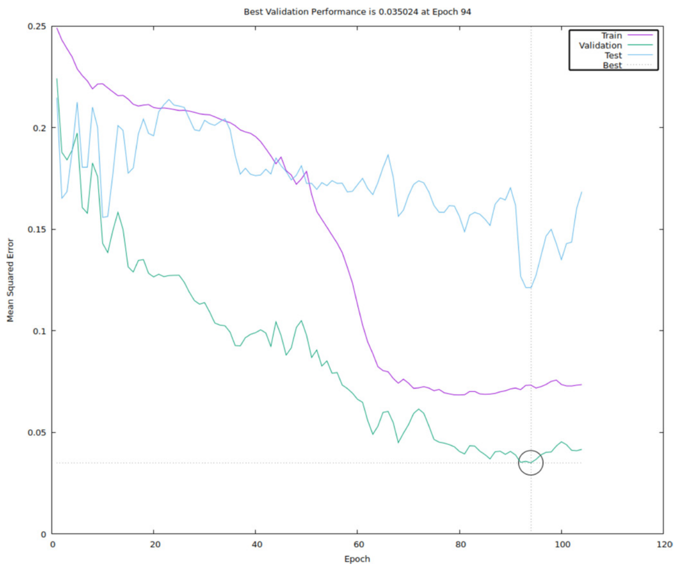

Supplementary Figure 2: Training, test and validation performance. Epoch by epoch, it is possible to see the MSE decay until the minimum global of validation, which is the stopping criterion in the early stopping regularization - needed to avoid overfitting.

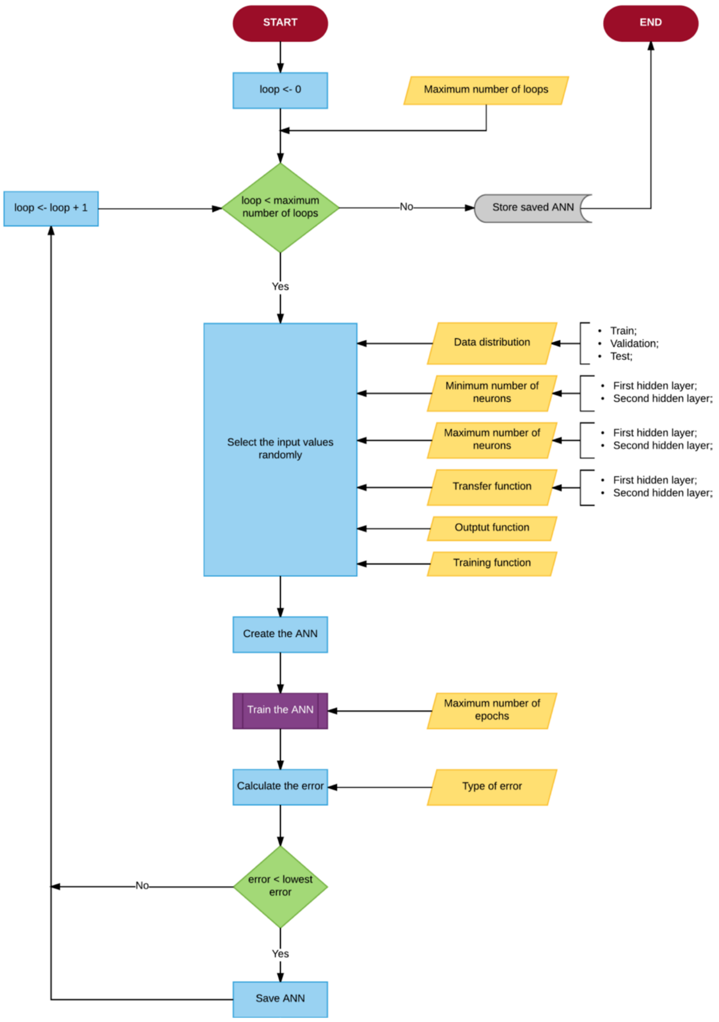

Supplementary Figure 3: Flowchart for the algorithm used to determine the best ANN topology.

Supplementary Table 1: Differentially represented miRNAs in plasma samples of controls and cancer patients

miRNAs	*p*-value	FR	miRNAs	*p*-value	FR
miR-1281	0.0011	–1.42	miR-92b-3p	0.0034	1.22
miR-489-3p	0.0033	–1.33	miR-16-2-3p	0.0047	1.23
miR-380-5p	0.0188	–1.29	miR-451a	0.0108	1.23
miR-532-3p	0.0032	–1.29	miR-18a-5p	0.0168	1.25
miR-3159	0.0028	–1.24	miR-25-3p	0.0047	1.25
miR-661	0.0011	–1.23	miR-22-3p	0.0083	1.27
miR-490-3p	0.0064	–1.21	miR-3614-3p	0.0478	1.27
miR-615-5p	0.0236	–1.20	miR-16-5p	0.0035	1.27
miR-1287-5p	0.0425	–1.19	miR-484	0.0032	1.28
miR-22-5p	0.0389	1.13	miR-140-3p	0.0232	1.28
miR-30a-5p	0.0299	1.14	miR-195-5p	0.0061	1.28
miR-378g	0.0465	1.14	miR-4755-3p	0.0003	1.29
miR-7-5p	0.0348	1.14	miR-363-3p	0.0009	1.30
miR-15b-3p	0.0198	1.15	miR-130a-3p	0.0392	1.30
miR-21-5p	0.0472	1.17	miR-185-5p	0.0055	1.31
let-7f-5p	0.0169	1.17	miR-186-5p	0.0019	1.31
miR-19b-3p	0.0432	1.17	miR-4796-5p	0.0222	1.31
miR-15b-5p	0.0299	1.19	miR-320a	0.0224	1.32
miR-29b-3p	0.0285	1.19	miR-425-5p	0.0036	1.32
miR-652-3p	0.0065	1.19	miR-29c-3p	0.0014	1.33
miR-92a-3p	0.0051	1.20	miR-15a-5p	0.0050	1.37
miR-17-3p	0.0049	1.20	miR-424-5p	0.0021	1.38
miR-19a-3p	0.0267	1.21	miR-4646-5p	0.0020	1.39
miR-590-5p	0.0036	1.21	miR-3679-5p	0.0011	1.40
miR-130b-3p	0.0064	1.21	let-7i-5p	0.0005	1.41
miR-18a-3p	0.0077	1.22	miR-4499	0.0070	1.43
miR-148a-3p	0.0458	1.22	miR-660-5p	0.0000	1.48
miR-4668-5p	0.0216	1.22	miR-101-3p	0.0003	1.50
			miR-144-3p	0.0003	1.53

Supplementary Table 2: Selected miRNA for validation

miRNAs	Fold regulation	*p* value	Set
hsa-miR-15a-5p	1,60	0,0016	I
hsa-miR-144-3p	1,61	0,0014	I
hsa-miR-101-3p	1,68	0,0006	I
hsa-miR-124-3p	1,94	0,0326	I
hsa-miR-3614-3p	1,71	0,0221	II
hsa-miR-3150a-5p	1,79	0,0300	II
hsa-miR-4796-5p	1,63	0,0017	II
hsa-miR-3150b-5p	1,73	0,0330	II
hsa-miR-133b	–1,51	0,0024	II
hsa-miR-1825	–1,82	0,0009	II
hsa-miR-144-3p	1,53	0,0003	merge
hsa-miR-101-3p	1,50	0,0003	merge
hsa-miR-324-3p	1,64	0,1938	merge
hsa-miR-5008-3p	1,50	0,7468	merge

Fold regulation (2^{-dCt} of cancer/ 2^{-dCt} of control), p-value and the analysis set are described.

Supplementary Table 3: Most stables miRNA between cancer and control from the merge analysis

	Control dCt	Cancer dCt	max-min
hsa-miR-328-3p	–3.25	–3.02	–3.02
hsa-miR-489-3p	0.02	0.43	–0.41
hsa-miR-21-5p	–1.41	–1.64	–0.23
hsa-miR-1280	*1.14*	*1.15*	*0.01*
hsa-miR-3173-3p	*0.25*	*0.26*	*0.01*
hsa-miR-3190-3p	0.70	0.72	0.03
hsa-miR-320b	1.99	1.95	0.04
hsa-miR-4659b-5p	1.84	1.91	0.06
hsa-miR-4746-3p	0.15	0.08	0.07
hsa-miR-4769-5p	1.08	1.18	0.09
hsa-miR-323b-3p	2.41	2.53	0.12
hsa-miR-30d-5p	1.99	1.86	0.13
hsa-miR-30e-5p	0.54	0.41	0.13
hsa-miR-637	–2.21	–2.07	0.15
hsa-miR-1238-3p	1.39	1.54	0.15
hsa-miR-3154	1.81	1.97	0.16
hsa-miR-30a-5p	0.72	0.55	0.17
hsa-miR-7-2-3p	0.07	0.27	0.20
hsa-miR-3201	1.39	1.19	0.21
hsa-miR-486-5p	–2.87	–3.08	0.21
hsa-miR-1260a	2.11	2.35	0.23
hsa-miR-766-3p	1.40	1.63	0.23
hsa-miR-628-3p	0.49	0.73	0.25
hsa-miR-675-3p	2.68	2.42	0.27
hsa-miR-92a-3p	–2.03	–2.30	0.27
hsa-miR-19a-3p	–0.88	–1.15	0.27
hsa-miR-490-3p	–2.67	–2.40	0.27
hsa-miR-661	2.46	2.76	0.30
hsa-miR-451a	–6.89	–7.19	0.30
hsa-miR-16-5p	–3.95	–4.31	0.36
hsa-miR-195-5p	–3.16	–3.52	0.36
hsa-miR-532-3p	1.74	2.11	0.36
hsa-miR-380-5p	0.87	1.23	0.37
hsa-miR-144-3p	0.05	–0.56	0.61

The lower the difference between delta Ct, the most stable the gene is.

Supplementary Table 4: Patients characteristic for the screening and validation phase

Error from training	9.37%
Error for validation	0.00%
Error for test	9.09%
Error for all data	7.54%
Mean Squared Error (MSE)	0.075163
Training data	60%
Validation data	20%
Test data	20%
Number of neurons in the first hidden layer	4
Number of neurons in the second hidden layer	5
Number of neurons in the output layer	1
Function in the first hidden layer	Sigmoid Symmetric
Function in the second hidden layer	Sigmoid Symmetric
Function in the output layer	Sigmoid
Training function	RPROP

*correspond to % of malignant BI-RADS 4 patients, 66% of all BI-RADs 4 patients were benign.

Supplementary Table 5: ANN reassessment about the train data

Output	Target	Error (Target - Output)	Prediction	Classification
0.013536	0	–0.013536	0	Correct
0.90035	1	+0.099650	1	Correct
0.897719	1	+0.102281	1	Correct
0.876149	1	+0.123851	1	Correct
0.264338	0	–0.264338	0	Correct
0.026153	0	–0.026153	0	Correct
0.067552	0	–0.067552	0	Correct
0.73639	1	+0.263610	1	Correct
0.773962	1	+0.226038	1	Correct
0.407973	0	–0.407973	0	Correct
0.885653	1	+0.114347	1	Correct
0.408019	1	+0.591981	0	Incorrect
0.013301	0	–0.013301	0	Correct
0.335433	1	+0.664567	0	Incorrect
0.029142	0	–0.029142	0	Correct
0.050491	0	–0.050491	0	Correct
0.888186	1	+0.111814	1	Correct
0.7456	1	+0.254400	1	Correct
0.773953	1	+0.226047	1	Correct
0.209411	0	–0.209411	0	Correct
0.407563	0	–0.407563	0	Correct
0.765531	0	–0.765531	1	Incorrect
0.013412	0	–0.013412	0	Correct
0.013185	0	–0.013185	0	Correct
0.22291	0	–0.222910	0	Correct
0.193798	0	–0.193798	0	Correct
0.177792	0	–0.177792	0	Correct
0.012975	0	–0.012975	0	Correct
0.013801	0	–0.013801	0	Correct
0.013652	0	–0.013652	0	Correct
0.78918	1	+0.210820	1	Correct

If Output > = 0.5, then Prediction = 1; else Prediction = 0. Benign lesion = 0 and Malignant lesion = 1.

Supplementary Table 6: ANN reassessment about the validation data

Output	Target	Error (Target - Output)	Prediction	Classification
0.013852	0	–0.013852	0	Correct
0.013858	0	–0.013858	0	Correct
0.309527	0	–0.309527	0	Correct
0.013846	0	–0.013846	0	Correct
0.773963	1	+0.226037	1	Correct
0.977441	1	+0.022559	1	Correct
0.012817	0	–0.012817	0	Correct
0.405978	0	–0.405978	0	Correct
0.192567	0	–0.192567	0	Correct
0.013823	0	–0.013823	0	Correct

If Output > = 0.5, then Prediction = 1; else Prediction = 0. Benign lesion = 0 and Malignant lesion = 1.
